# Integrated In Silico, In Vitro, and In Vivo Studies Reveal Mangiferin as a Promising Antiviral Agent Against H1N1/pdm2009 Influenza Virus

**DOI:** 10.3390/v17070873

**Published:** 2025-06-21

**Authors:** Yinde Gan, Fucheng Guo, Ayan Roy, Xiao Wang, Yongyi Shen

**Affiliations:** 1 State Key Laboratory for Animal Disease Control and Prevention, Center for Emerging and Zoonotic Diseases, College of Veterinary Medicine, South China Agricultural University, Guangzhou 510642, China; coocooing41@gmail.com (Y.G.); guofucheng@gdou.edu.cn (F.G.); 17328461020@163.com (X.W.); 2 College of Coastal Agricultural Science, Guangdong Ocean University, Zhanjiang 524088, China; 3 Department of Biological Sciences, Asian University for Women, Chittagong 4000, Bangladesh; ayanroy.bio@gmail.com; 4 School of Agriculture and Biology, Shanghai Jiao Tong University, Shanghai 200240, China

**Keywords:** mangiferin, influenza virus A(H1N1)pdm09, molecular docking

## Abstract

The ongoing global threat posed by the influenza A virus, exacerbated by antigenic drift and the emergence of antiviral resistance, accentuates the urgent need for innovative therapeutic strategies. Through molecular docking, this study revealed that mangiferin has a strong binding affinity for the active site of the neuraminidase (NA) protein of influenza virus A(H1N1)pdm09, with a binding energy of −8.1 kcal/mol. In vitro assays confirmed a dose-dependent inhibition of NA, with an IC_50_ of 88.65 μM, and minimal cytotoxicity, as indicated by a CC_50_ of 328.1 μM in MDCK cells. In murine models, the administration of mangiferin at a dosage of 25 mg/kg significantly mitigated weight loss, decreased viral loads in nasal turbinates and lungs by over 1 log10 TCID_50_, and enhanced survival rates from 0% in control groups to 20% in mangiferin-treated group at 14 days post-infection. In addition, mangiferin was found to modulate host immune responses by simultaneously inhibiting pro-inflammatory cytokines, IL-6 and TNF-α, and upregulating the expression of anti-inflammatory IL-10 and antiviral IFN-γ, thus mitigating infection-induced inflammation. Our findings elucidate the dual mechanism of mangiferin involving the direct inhibition of NA and immunomodulation, thereby providing experimental evidence for exploring dual-mechanism-based anti-influenza strategies against resistant strains of influenza.

## 1. Introduction

The influenza virus A(H1N1)pdm09 culminated in the first devastating influenza pandemic of the 21st century, resulting in over 18,500 deaths globally and perpetuating seasonal epidemics [1,2]. This distinctive triple-reassortant virus exhibits significant pathogenicity in the human population in diverse age groups, particularly affecting young adults who were previously considered low risk [3]. Moreover, its continuous evolution into multiple antigenically distinct subclades has complicated vaccine development and has significantly challenged the existing antiviral therapies [3,4].

Neuraminidase (NA), a critical glycoprotein located on the surface of the H1N1 virus, possesses an unique “150-cavity” structural motif within its tetrameric configuration, which serves as a basis for targeted drug development [5]. The advantages of NA as a therapeutic target include the structural conservation of its active site, precise three-dimensional resolution, an expanded therapeutic window, and a reduced antigenic variation compared to the H1N1 hemagglutinin [6]. 

However, neuraminidase inhibitors are currently encountering resistance due to mutations at the active site, such as H274Y and E119V [7,8,9]. Furthermore, the influenza virus A(H1N1)pdm09 has developed an intrinsic resistance to adamantanes through the S31N substitution [10], underscoring the pressing need for novel therapeutics and innovative treatment approaches.

Computational approaches, relying on virtual screening of small molecules, molecular docking, and molecular dynamics simulations, have emerged as powerful tools in antiviral drug discovery. Molecular docking provides detailed insights into ligand–receptor binding conformations, simultaneously facilitating the robust and cost-effective identification of drug leads for experimental validation [11]. Computational studies have demonstrated that the R294K mutation in H7N9 neuraminidase confers oseltamivir resistance by altering the active site geometry, while maintaining zanamivir binding through distinct hydrogen bonding [12,13]. This capacity to analyze drug-resistant mutations has guided the optimization of next-generation inhibitors against oseltamivir-resistant influenza virus A(H1N1)pdm09 that harbors the H275Y mutation. Traditional Chinese medicine (TCM) has been effectively used for centuries to treat viral infections [14,15]. Bioactive compounds in TCM have been reported to demonstrate significant antiviral activities against a wide range of viruses, including influenza [16,17,18]. A recent study identified bioactive compounds from *Scutellaria baicalensis* that inhibit influenza virus proteins, revealing binding modes analogous to clinically validated antiviral drugs [19]. However, there are relatively few studies that have leveraged molecular docking techniques towards a large-scale screening of TCM-derived small-molecule drug leads against A(H1N1)pdm09 neuraminidase.

This study evaluated the inhibitory potential of TCM-derived compounds—structurally diverse plant natural products including terpenoids, alkaloids, and phenolic compounds with traditional medicinal properties such as antiviral and anti-inflammatory effects—against the neuraminidase of influenza virus A(H1N1)pdm09, aiming to identify candidate inhibitors targeting this protein. Our results based on in silico, in vitro, and in vivo investigations reveal mangiferin as a promising inhibitory candidate. Mangiferin, a C-glycosyl flavonoid extracted from plants such as Mangifera indica, displays a range of pharmacological activities, including antioxidant, anti-inflammatory, and immunomodulatory effects [20]. Preliminary investigations have indicated its inhibitory potential against several viruses, including influenza [21,22]. However, the specific mechanisms by which mangiferin exerts its inhibitory effects against influenza virus A(H1N1)pdm09, particularly through its interaction with neuraminidase and its immunomodulatory properties, have not yet been elucidated. Therefore, this study aims to explore the antiviral activity of mangiferin against influenza virus A(H1N1)pdm09 through a combination of computational simulations and experimental validations. 

## 2. Materials and Methods

### 2.1. Molecular Docking

The high-resolution crystal structure of the A(H1N1)pdm09 neuraminidase (NA) protein (PDB ID: 4B7Q Chain A) [5] was retrieved from PDB (http://www.rcsb.org). AutoDockTools was used to prepare the protein structure by removing water and inhibitor molecules, adding polar hydrogen atoms, and assigning Kollman charges. Energy minimization was achieved through the YASARA energy minimization server [23]. 

The SMILES structures of the TCM-derived small-molecule ligands and the two drugs (oseltamivir and zanamivir) were retrieved from the NCBI PubChem database (https://pubchem.ncbi.nlm.nih.gov/), accessed on 15 September 2024. The corresponding three-dimensional structures were generated using the Open Babel software [24]. The ligand structures were energy-minimized using the PRODRG server [25]. Gasteiger charges were added to the ligands using AutoDockTools, prior to molecular docking. 

Molecular docking was performed using AutoDock Vina [26] opting a grid-based approach. The protocol employed a rigid protein receptor and flexible ligand docking methodology. The ligands were docked at the inhibition pocket of A(H1N1)pdm09 neuraminidase protein with prior information about its active site [5], using a suitable grid box (center x = 21.563, center y = 22.474, center z = −30.509, size x = 50.75, size y = 50.75, size z = 50.75). The binding energies (ΔG, kcal/mol) were determined based on the lowest-energy docking conformations. Since AutoDock Vina employs deterministic algorithms for energy calculations, the binding energy values represent direct software outputs that show minimal variation across independent runs (typically < 0.1 kcal/mol), making confidence intervals unnecessary for these computational predictions. The molecular interactions of the protein–ligand complexes, which included hydrogen bonds, hydrophobic interactions, and steric complementarity, were visualized through the Discovery Studio Visualizer (version = v25.1).

### 2.2. Assessment of Mangiferin’s Anti-Neuraminidase Activity

The inhibitory efficacy of mangiferin on the neuraminidase (NA) activity of A(H1N1)pdm09 was assessed using the neuraminidase inhibitor screening kit (Beyotime Biotechnology, Shanghai, China), in accordance with the manufacturer’s instructions.

### 2.3. Virus Strain and Propagation

The influenza virus A(H1N1)pdm09 (A/Guangzhou/03/2009), with an EC_50_ of 6.72 μM in MDCK cells [27], was generously provided by Professor Jianxin Chen from the College of Veterinary Medicine at the South China Agricultural University. Current GISAID records indicate A(H1N1)pdm09/6B.1A.5a.2a.1 as the dominant human influenza strain. The comparative analysis of its NA sequence (EPI ISL 19891295) revealed 98.3% identity with our isolate. Viral propagation was conducted in 9- to 11-day-old specific-pathogen-free (SPF) chicken embryos. The virus was inoculated into the allantoic cavity of the chicken embryos and incubated at 37 °C for 48 h. Allantoic fluid was collected and the presence of the virus was confirmed through a hemagglutination (HA) assay. For HA assay, 50 μL of culture supernatant was mixed with 50 μL of 1% chicken red blood cell suspension in V-bottom 96-well plates, incubated at room temperature for 25 min, and examined for hemagglutination. 

### 2.4. Virus Titration

Viral titers were calculated using 50% tissue culture infectious dose (TCID_50_). MDCK cells (ATCC CCL-34) were maintained in Dulbecco’s Modified Eagle Medium (DMEM, Gibco, Grand Island, NY, USA) supplemented with 10% fetal bovine serum (FBS, Gibco), 100 U/mL penicillin, and 100 μg/mL streptomycin at 37 °C in a 5% CO_2_ atmosphere.

Virus titration was performed on MDCK cells. MDCK cells were seeded at a density of 1 × 10^4^ cells per well in 96-well plates. Once confluent, ten-fold serial dilutions (from 10^−1^ to 10^−8^) of the A(H1N1)pdm09 virus were added. After 48 h of incubation, viral infectivity was assessed through a hemagglutination (HA) assay, and TCID_50_ values were calculated using the Reed–Muench method [28]. All procedures involving live viruses were conducted in accordance with biosafety level 2 (BSL-2) containment protocols.

### 2.5. Cytotoxicity Assessment of Mangiferin

The cytotoxic effects of mangiferin were assessed on Madin–Darby Canine Kidney (MDCK) cells using the 3-(4,5-dimethylthiazol-2-yl)-2,5-diphenyltetrazolium bromide (MTT) assay. In brief, MDCK cells were harvested when they reached 80–90% confluence, subjected to trypsinization, and were subsequently plated into 96-well plates at a density of 1 × 10^4^ cells/mL (100 μL per well). To mitigate edge effects, peripheral wells were filled with 200 μL of phosphate-buffered saline (PBS). Mangiferin was solubilized in dimethyl sulfoxide (DMSO) to create a 10 mM stock solution. Subsequently, it was serially diluted in Dulbecco’s Modified Eagle Medium (DMEM) that was supplemented with 1% penicillin–streptomycin and 0.2% bovine serum albumin (BSA) to establish eight concentration gradients (640, 320, 160, 80, 40, 20, and 10 μM). Following a 48-hour treatment period, the supernatant was removed, and 100 μL of a 0.5 mg/mL MTT solution was introduced to each well. After a 4-hour incubation at 37 °C, the MTT solution was discarded, and 150 μL of DMSO was added to solubilize the formazan crystals. The plates were then agitated on a shaker at 37 °C for 10–15 min, and the optical density (OD) was measured at 490 nm using a microplate reader. Cell viability was calculated as:

$$Cell\;Viability\left(\%\right)=\left(\frac{{OD}_{treatment}}{{OD}_{negativecontrol}}\right)\times 100$$

### 2.6. Evaluation of Mangiferin’s Antiviral Activity Against Influenza Virus A(H1N1)pdm09

MDCK cells were plated into 12-well plates at a density of 1 × 10^5^ cells/mL and cultured for 24 h until they reached 80–90% confluence. The culture medium was aspirated and the cells were washed twice with 2 mL of PBS per well. Subsequently, the H1N1/2009 virus, diluted to 100 TCID_50_ (1 mL per well), was added to infect the cells, while the blank control group received 1 mL of DMEM containing 1% penicillin–streptomycin and 0.2% BSA. After allowing for 1–2 h of viral adsorption, the cells were treated with mangiferin (diluted in 0.2% BSA, 1 µg/mL TPCK-treated trypsin, 1% penicillin–streptomycin, and serum-free DMEM) at concentrations of 80, 40, 20, 10, and 5 μM. Zanamivir (2 μM) was utilized as a positive control, while the negative control group received only the dilution buffer. Additionally, a virus- and drug-free blank group was included in the experimental design. Following a 48-hour incubation period at 37 °C, the supernatants were collected, and viral titers were quantified using the TCID_50_ method. Each treatment group, including the controls, was evaluated in triplicate.

### 2.7. In Vivo Assessment of Mangiferin’s Efficacy Against Influenza Virus A(H1N1)pdm09

Due to ethical considerations and the 3Rs principle (Replacement, Reduction, and Refinement), we limited animal groups to essential comparisons. The efficacy of NA inhibitors in this model has been well-established [29]. Female C57BL/6J mice, aged six to eight weeks, were housed in pathogen-free isolators following protocols approved by the Institutional Animal Care and Use Committee at South China Agricultural University (Approval No. 2025C042). They were acclimatized and were subsequently allocated into four distinct groups (*n* = 15 per group): control group, which received intranasal administration of 50 µL phosphate-buffered saline (PBS) alongside oral gavage of PBS containing 2% dimethyl sulfoxide (DMSO) at a dosage of 5 mL/kg; mangiferin-only group, which received intranasal PBS and oral mangiferin at a dosage of 25 mg/kg in PBS with 2% DMSO; H1N1-infected group, which underwent intranasal inoculation with 50 μL of influenza virus A(H1N1)pdm09 (10^5^ TCID_50_) and oral PBS with 2% DMSO; and mangiferin-treated group, which received both intranasal virus inoculation and oral mangiferin at a dosage of 25 mg/kg. Anesthesia was administered to facilitate intranasal delivery, and treatments were provided via oral gavage every 12 h over a period of six days.

Clinical monitoring and sample collection involved the daily recording of body weight and survival rates. Mice from the H1N1-infected and mangiferin-treated groups were euthanized on days 3, 6, and 9 post-infection (dpi), with serum, lung tissues, and nasal turbinates being collected for analysis. Lung tissues designated for histological examination were fixed in 4% paraformaldehyde.

For viral titration in tissues, nasal turbinates and lung tissues were homogenized in Dulbecco’s Modified Eagle Medium (DMEM) containing antibiotics (0.2 g tissue/mL) utilizing a tissue homogenizer (30 Hz, 3 min × 3 cycles). The homogenates were then centrifuged at 13,000× *g* for 5 min at 4 °C. Supernatants were serially diluted (10^−1^ to 10^−8^) in DMEM, and 200 μL of each dilution was inoculated into the allantoic cavity of three 9–11-day-old specific pathogen-free (SPF) chicken eggs. The eggs were incubated at 37 ± 0.5 °C with 55–65% humidity for 72 h. Viral titers (log10 TCID_50_/g tissue) were assessed using a hemagglutination (HA) assay and were calculated using the Reed–Muench method.

For RNA extraction and cytokine profiling, total RNA from lung tissues was isolated using the TransZol Up Kit (TransGen Biotech, Beijing, China). First-strand cDNA synthesis was conducted with the TIANScript II cDNA Synthesis Kit (TIANGEN Biotech, Beijing, China). Cytokine mRNA levels (IL-6, IL-10, TNF-α, and IFN-γ) were quantified through quantitative polymerase chain reaction (qPCR) utilizing ChamQ Universal SYBR qPCR Master Mix (Vazyme Biotech, Nanjing, China) on a QuantStudio 5 Real-Time PCR System (Thermo Fisher Scientific, Waltham, MA, USA). The primer sequences are detailed in Supplementary Table S1.

### 2.8. Statistical Analysis

Data are presented as mean ± standard error of the mean (SEM). Survival curves were evaluated using the Kaplan–Meier method accompanied by log-rank tests. Comparisons of viral titers and cytokine levels were performed using the two-way analysis of variance (ANOVA), followed by Tukey’s post hoc test (GraphPad Prism 10.0). A *p*-value of less than 0.05 was considered statistically significant. All computational analyses were executed with default parameters, unless otherwise indicated.

## 3. Results

### 3.1. Molecular Docking Analysis Revealed Mangiferin as a Promising Inhibitory Candidate Against Influenza Virus A(H1N1)pdm09 Neuraminidase

Molecular docking was performed to evaluate the inhibitory potential of TCM-derived compounds and the drugs oseltamivir and zanamivir against the NA protein of influenza virus A(H1N1)pdm09. The top binding energy scores are detailed in Table 1. 

**Figure 1:**
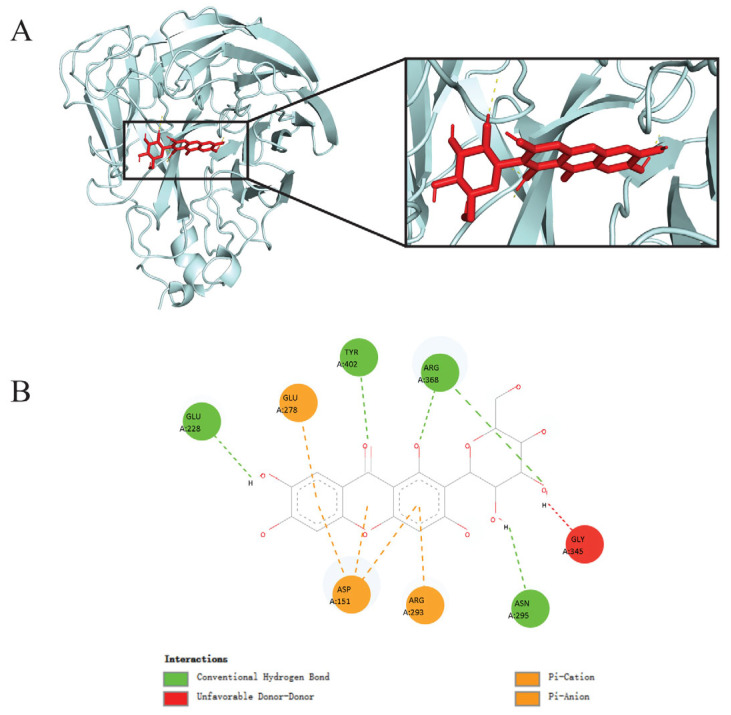
Interaction of mangiferin with A(H1N1)pdm09 neuraminidase protein revealed through molecular docking. (**A**) Mangiferin (red) bound neuraminidase (silver) at its active binding site. (**B**) Binding mode of mangiferin with neuraminidase revealing the interacting amino acid residues and bonds at the active binding site of neuraminidase.

Among the TCM-derived compounds, mangiferin demonstrated the most promising inhibitory potential, with a binding energy score of −8.1 kcal/mol against the NA protein (Table 1). Interestingly, mangiferin displayed a stronger binding affinity than the standard antiviral drugs oseltamivir (−8.0 kcal/mol) and zanamivir (−6.4 kcal/mol). A thorough interaction profiling (Figure 1) revealed that mangiferin interacted with the NA protein at its active site involving the amino acid residues Glu228, Asn295, Arg368, and Tyr402 through hydrogen bonds (Figure 1B). In addition, mangiferin was found to interact through π-cations and π-anions with the Asp151, Glu278, and Arg293 residues of the NA protein (Figure 1B). Our data provide encouraging evidence that mangiferin promises to be a potential influenza virus neuraminidase inhibitor, warranting the further validation of its therapeutic potential in vitro and in vivo.

### 3.2. Mangiferin’s Anti-Neuraminidase Activity and Cytotoxicity In Vitro

**Figure 2:**
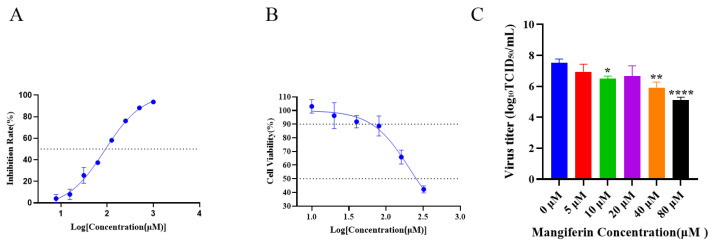
Mangiferin’s anti-neuraminidase activity and cytotoxicity in vitro. (**A**) Dose–response curve of mangiferin on neuraminidase inhibition (IC_50_ = 88.65 μM; R^2^ = 0.9898). (**B**) Evaluation of mangiferin cytotoxicity using the MTT assay (R^2^ = 0.8426). (**C**) Viral titers of influenza virus A(H1N1)pdm09 treated with different mangiferin concentrations (Student’s *t*-test was used to assess statistical significance, compared to 0 μM; * indicates *p* < 0.05, ** indicates *p* < 0.01; **** indicates *p* < 0.0001).

To investigate the inhibitory effects of mangiferin on neuraminidase (NA), a concentration-dependent binding and inhibition assay was conducted. The results, illustrated in Figure 2A, indicate that mangiferin exhibits a concentration-dependent inhibition of NA activity, with an IC_50_ (half maximal inhibitory concentration) value of 88.65 μM. 

To evaluate the cytotoxicity of mangiferin, MDCK cells were treated with mangiferin at concentrations ranging from 0 to 320 μM. The results demonstrate concentration-dependent cytotoxic effects, as shown in Figure 2B. Mangiferin concentrations ≤60.01 μM showed no significant cytotoxicity towards MDCK cells, with cell viability maintained at 90% (Figure 2B). The cytotoxic concentration 50% (CC_50_) was found to be 328.1 μM.

Given the low cytotoxicity threshold (≤60.01 μM) and NA inhibitory activity (IC_50_ = 88.65 μM), the anti-influenza activity of mangiferin against the H1N1/pdm 2009 virus was evaluated at concentrations up to 80 μM. Concentration-dependent antiviral effects were observed, with significant viral replication inhibition being detected at ≥10 μM (Figure 2C). Zanamivir (5 μM), employed as a positive control, effectively suppressed viral replication under identical experimental conditions. Mangiferin at 80 μM achieved a 2.4 log reduction in viral titer, meeting the threshold for biological significance (≥2 log reduction). However, this effective concentration approached the IC_50_ value (88.65 μM), thus highlighting the need for structural optimization to improve potency.

### 3.3. Effects of Mangiferin on Influenza Virus A(H1N1)pdm09 in a Murine Model

**Figure 3:**
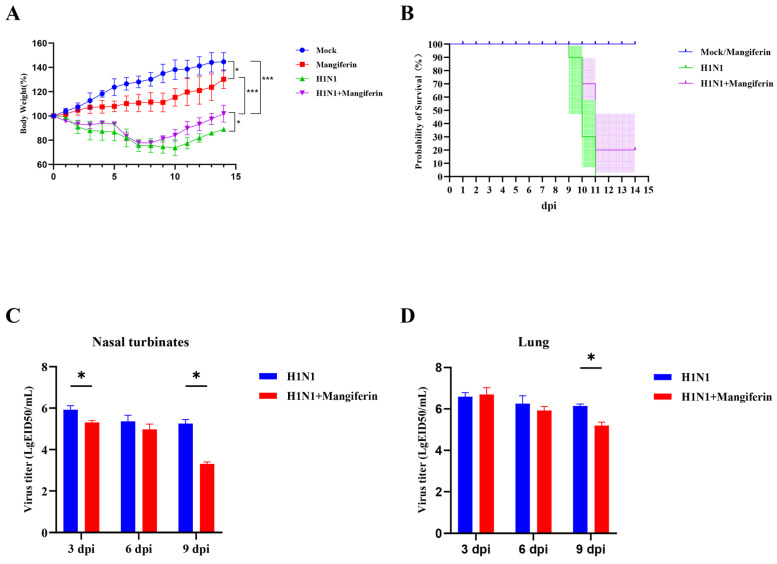
Clinical observations across different experimental groups of mice. (**A**) Changes in body weight using two-way ANOVA. (**B**) Survival rates by log-rank test (χ^2^ = 20.51, *p* < 0.0001), with a significant trend across groups (*p* = 0.0018). (**C**) Viral titer in nasal turbinates. (**D**) Viral titer in lungs. In (**C**,**D**), statistical significance was determined by unpaired multiple *t*-tests. * *p* < 0.05; *** *p* < 0.001.

In our study, the mice in the control group (PBS via gavage) and mangiferin-only group (mangiferin dissolved in PBS via gavage) exhibited a sustained gain in body weight throughout the experimental period. In contrast, the H1N1 virus-infected mice group showed a 60% reduction in body weight compared to the baseline, indicating a trend toward metabolic impairment associated with viral infection. The mangiferin-treated H1N1-infected mice group displayed a modest reduction in weight loss compared to the H1N1-infected group without mangiferin treatment (Figure 3A).

Both the control and mangiferin-only groups maintained a 100% survival rate throughout the 14-day post-infection (dpi) observation period (Figure 3B). The survival rate in the H1N1-infected group experienced a rapid decline from 90% to 30% from 9 to 11 dpi, culminating in total mortality by 11 dpi. Mangiferin treatment significantly improved survival (*p* < 0.0001 by log-rank test), with the group receiving mangiferin treatment demonstrating a survival rate of 70% at 10 dpi (contrasted to 40% in the H1N1-infected group) and stabilized at 20% from 11 to 14 dpi.

Viral titer assessments conducted in the nasal turbinates and lungs revealed peak viral loads at 3 dpi, followed by a gradual decline (Figure 3C,D). Treatment with mangiferin resulted in a significant (*p* < 0.05) reduction in viral titers when compared to the H1N1-infected cohort, with the inhibitory effects being more pronounced over time. 

**Figure 4:**
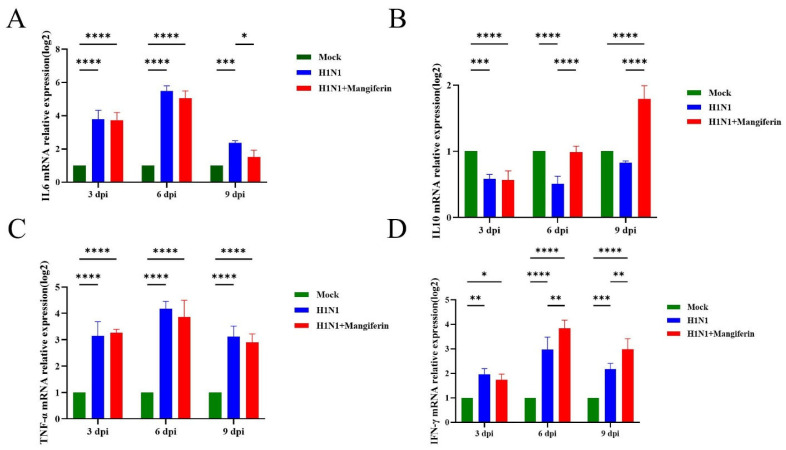
The relative expression levels of cytokines IL-6, IL-10, TNF-α, and IFN-γ in mice lung tissues across the three experimental mice groups—the control group (uninfected and untreated mice), the H1N1-infected group, and the H1N1-infected group treated with mangiferin. (**A**) IL-6; (**B**) IL-10; (**C**) TNF-α; (**D**) IFN-γ. Statistical significance was determined by s two-way ANOVA. * *p* < 0.05; ** *p* < 0.01; *** *p* < 0.001; **** *p* < 0.0001.

Utilizing mouse glyceraldehyde-3-phosphate dehydrogenase (GAPDH) as a reference, the relative expression levels of the cytokines IL-6, IL-10, TNF-α, and IFN-γ were estimated from the lung tissues across the three experimental mice groups—the control group (uninfected and untreated mice), the H1N1-infected group, and the H1N1-infected group treated with mangiferin. The expression of pro-inflammatory IL-6 exhibited a significant increase in the infected group at 3 dpi, peaking at 6 dpi. The mangiferin-treated group displayed significantly lower (*p* < 0.05) IL-6 expression levels compared to the infected group (Figure 4A). Anti-inflammatory IL-10 expression remained low in all groups, except the mangiferin-treated group that showed elevated levels at 9 dpi (Figure 4B). Pro-inflammatory TNF-α levels in the infected group increased rapidly at 3 dpi, peaked at 6 dpi, and slightly decreased by 9 dpi. On the contrary, the mangiferin-treated group exhibited lower TNF-α levels than the infected group starting from 6 dpi (Figure 4C). Antiviral cytokine IFN-γ expression in the infected group rose from 3 dpi but declined by 9 dpi. In contrast, the mangiferin-treated group maintained significantly higher (*p* < 0.01) levels of IFN-γ expression from 6 dpi onward (Figure 4D).

## 4. Discussion

Due to frequent antigenic drifts in influenza virus A(H1N1) pdm09, the antigenic epitopes of circulating strains undergo significant changes annually [30]. The licensed influenza vaccines are primarily focused on the antigenic characteristics of HA as the protective vaccine antigen. When vaccine strains are not aptly matched with the circulating viruses, gaps in population immunity barriers emerge, potentially leading to large-scale infections [31]. In this context, screening for medicine components with anti-influenza activity holds significant promise.

The evolutionary rate as determined by the accumulation of mutations over time is much lower for NA than for HA [32,33], and the NA is a better therapeutic target compared to the HA [6]. In this paper, we employed molecular docking to predict the binding affinities between a series of TCM monomeric compounds and the influenza virus A(H1N1) pdm09 NA protein. Our analysis identified mangiferin as a potential NA inhibitor. Mangiferin demonstrated a binding energy of −8.1 kcal/mol with the NA protein, surpassing the clinically established drug zanamivir (−6.4 kcal/mol, *p* < 0.0001). The interaction between mangiferin and NA suggests its potential antiviral mechanism. Molecular docking revealed that imperative active site residues Glu228, Asn295, Arg368, and Tyr402 form hydrogen bonds with the hydroxyl groups of mangiferin, exhibiting binding patterns similar to those of standard NA inhibitors such as zanamivir [34]. This observation suggests that mangiferin may competitively inhibit NA catalytic activity. Furthermore, Asp151, Glu278, and Arg293 interacted with mangiferin through π-cation and π-anion interactions, where the flexible 150-loop-containing Asp151 participated in conformational regulation [35]. This synergistic combination of hydrogen bonding and π-interactions resembles the mechanism of natural polyphenolic broad-spectrum inhibitors [36]. Compared to inhibitors relying on single interaction modes, such cooperative binding may confer a broad spectrum antiviral activity by circumventing resistance mutations (e.g., E119V) associated with conventional therapies [37]. The interaction strategy observed in mangiferin–NA binding highlights its potential as a structurally distinct antiviral agent with enhanced resistance to mutations and evolutionary adaptations of the influenza virus.

The inhibitory effects of mangiferin on influenza virus neuraminidase were evaluated in vitro. The results reveal that mangiferin exhibits a significant concentration-dependent inhibition, with a half-maximal inhibitory concentration (IC_50_) of 88.65 μM. In comparison, zanamivir demonstrated a median IC_50_ value of 0.42 nM (range: 0.1–3.43 nM) against the A(H1N1) pdm09 strain [38].

Mangiferin showed no significant impact on MDCK cell viability at a concentration of 60.01 μM (cell viability > 90%), with a CC_50_ (half-maximal cytotoxic concentration) of 328.1 μM. These findings suggest that mangiferin exhibits a relatively low cytotoxicity within its effective NA inhibitory concentration range (IC_50_ = 88.65 μM). Although mangiferin’s safety index (SI) (CC_50_/IC_50_ = 3.7) was found to be below the ideal threshold for conventional antivirals (e.g., zanamivir’s SI = 1.09 [39]), its efficacy in vivo at non-toxic doses (25 mg/kg) and immunomodulatory properties support its therapeutic potential. Further chemical modifications may enhance its SI [40]. Notably, mangiferin’s in vitro NA inhibitory activity (IC_50_ = 88.65 μM) is significantly weaker than the licensed NA inhibitors, supporting its clinical translation as a standalone antiviral agent, with further structural optimization and tailored delivery strategies. Our findings align with previous studies that reported negligible cytotoxicity of mangiferin in the CCD-25Lu normal human lung fibroblast cell line [41] and NCM460 normal human colon mucosal epithelial cell line [42]. Collectively, our data suggest that mangiferin holds significant potential as a safe novel therapeutic agent against influenza virus neuraminidase, with no harmful effect on normal human cell lines.

Earlier studies have demonstrated that mangiferin is therapeutically effective at high doses [43,44]. In contrast, in this paper, we demonstrate the substantial therapeutic efficacy of mangiferin at a significantly lower dosage of 25 mg/kg in mice. Our experiments in a murine model reveal that the treatment with mangiferin significantly enhances survival rates in H1N1-infected mice (Figure 3B), reduces lung tissue viral titers to one-tenth of control levels at later stages of infection (9 dpi) (Figure 3D), and does not result in any notable adverse toxic reactions (Figure 3A). Mangiferin, a naturally derived flavonoid, has earlier been reported to be pharmacokinetically and pharmacodynamically stable [45,46] and non-toxic, with no genotoxic or mutagenic effects in mammalian micronucleus [47] and murine models [48,49], at optimized doses. Although mangiferin exhibited effective NA inhibition in vitro, the murine model showed only a few therapeutical effects. The further modification of this compound is required. 

H1N1 infection is characterized by severe immune dysregulation consistent with inflammasome activation and cytokine storm [50,51]. Immune abnormalities in H1N1 infection trigger hypercytokinemia with an elevated expression of pro-inflammatory cytokines, including IL-6 and TNF-α [50]. In this paper, we investigated the immunomodulatory role of mangiferin in H1N1-infected mice. Our results demonstrate that, apart from arresting viral replication, mangiferin treatment significantly decreases the expression of pro-inflammatory cytokines IL-6 and TNF-α in the lung tissue of mice (refer to Figure 4A,C). Our data are consistent with previous studies that reported the mangiferin-induced suppression of IL-6 and TNF-α in mice [44,52] and human lymphocytes [53,54]. Studies have suggested that mangiferin mitigates inflammation by inhibiting NF-κB and NLRP3 inflammasome signaling [44,54]. Concurrently, mangiferin treatment upregulated the expression levels of the anti-inflammatory cytokine IL-10 and the antiviral immune factor IFN-γ (Figure 4B,D). Previous studies have demonstrated that the concentration-dependent mangiferin treatment induces IL-10 and IFN-γ expression in mice [55] and upregulates IL-10 levels in human macrophages [56] and lymphocytes [53]. This dual immunomodulatory action of mangiferin is notably uncommon among natural monomeric compounds and contrasts with that of the traditional Chinese medicine formula KBD, which activates the MAVS pathway to enhance IFN-β expression [57]. Although KBD includes mangiferin, its multi-component synergistic mechanism may alter the specific effects of individual constituents [57]. In this paper, we provide compelling evidence for the immnunoprotective and immunomodulatory roles of mangiferin, apart from its antiviral properties. Despite limited direct NA inhibition, mangiferin’s immunomodulatory effects demonstrated unique value in vivo: attenuated lung inflammation at 25 mg/kg (Figure 4) and improved late-stage survival (Figure 3B). This supports its potential as an adjuvant therapy to complement conventional NA inhibitors.

In conclusion, based on integrated in silico molecular docking and comprehensive experimental investigations, our study reports the effective inhibitory of mangiferin against influenza virus A(H1N1)pdm09 neuraminidase. Animal challenge experiments reveal the protective role of mangiferin against influenza virus A(H1N1) pdm09. Our findings highlight the synergistic antiviral and anti-inflammatory role of mangiferin. Future studies should focus on structurally optimizing mangiferin to enhance its bioactivity and exploring its combination with immunomodulatory agents, enabling multi-target strategies against influenza infection.

## Figures and Tables

**Table 1:** Binding energy scores of TCM-derived compounds and antiviral drugs with A(H1N1)pdm09 neuraminidase protein. Data represent mean of three independent docking replicates.

Ligands	Binding Energy (kcal/mol)
Mangiferin	−8.1
Oseltamivir (Drug)	−8.0
Isobellidifolin	−7.6
Swerchirin	−7.6
Swertianin	−7.6
Trachylobane	−7.4
Anisotine	−7.3
Gentiopicrin	−7.3
Adhatodine	−7.3
Lupeol	−7.2
Stigmasterol	−7.2
Betulin	−7.0
Beta-sitosterol	−6.9
Decussatin	−6.8
Vasicoline	−6.6
Clerosterol	−6.6
Zanamivir (Drug)	−6.4
Caryophyllene	−6.4
Vasicine	−6.3
Gentianine	−6.0
Enicoflavine	−6.0
Carvacrol	−5.8
Cineole	−5.6
Eugenol	−5.5
Squalene	−5.2
Catechol	−5.1

## Data Availability

The datasets generated and/or analyzed during the current study are available from the corresponding author upon reasonable request.

## Supplementary Materials

The following supporting information can be downloaded at: https://www.mdpi.com/article/10.3390/v17070873/s1, Table S1: Primer list for cytokine detection.
