# Supplementary material for: Integrated In Silico, In Vitro, and In Vivo Studies Reveal Mangiferin as a Promising Antiviral Agent Against H1N1/pdm2009 Influenza Virus
Source: Viruses. 2025 Jun 21;17(7):873. doi: 10.3390/v17070873 (PMC12299334; doi:10.3390/v17070873)
Supplement: Supplementary file 1 [file viruses-17-00873-s001.zip › viruses-3665273-supplementary.pdf]

Supplementary Materials

Table S1: Primer list for cytokine detection.

Primer Name	Primer sequences （5′→3′）
IL6-F	GCCAGAGTCCTTCAGAGAGATACAG
IL6-R	GTCCTTAGCCACTCCTTCTGT
IL10-F	GCAGGACTTTAAGGGTTACTTGGG
IL10-R	CTCACCCAGGGAATTCAAATGCTC
TNF-α-F	CCCAGACCCTCACACTCAGATC
TNF-α-R	CACTCCAGCTGCTCCTCCAC
IFN-γ-F	ACTACCTTCTTCAGCAACAGCAAG
IFN-γ-R	TGAATGCTTGGCGCTGGAC
